# On Creosote as a Remedy for Deafness

**Published:** 1840-05-30

**Authors:** O. H. Partridge

**Affiliations:** Philadelphia


### ON CREOSOTE AS A REMEDY FOR DEAFNESS.

BY O. H. PARTRIDGE, M. D.

From numerous observations that I have made in hospital and private practice, I believe that four cases out of five of deafness are caused either from local debility, producing what is generally called "nervous deafness," or from a want of action in the ceruminous glands, or in consequence of the external passage becoming obstructed from wax, mucus, or some foreign substance getting into the ear.

I lay no claims to originality, in the course of treatment I have been in the practice of pursuing, but will merely state facts as they have come under my notice, with the wish, if others have not prescribed the same course of treatment, that they will give it a fair trial. A large number of cases of morbid hearing, and some of long standing, have come under my observation within the last three or four years, and when they were produced by the above mentioned causes, I have generally been successful in curing, or greatly relieving, the patients. My directions are as follows: to have the meatus auditorius thoroughly cleansed, I cause to be dropped into the ear night and morning for five or six days, a few drops of olive oil or the oil of almonds, and injecting with a very small syringe, once a day, a solution of the best castile soap in warm water, with a little eau de cologne, in the proportions of six parts of the solution, to one of the cologne. When on examination I find the ear perfectly clean, I then commence with the creosote, which, I think, will act more speedily and efficaciously by stimulating and producing a healthy action in the parts diseased, than any application I have ever yet seen used. I commence with the following formula:

℞.—Creosote, ʒss.

Ol. Amygdalæ, ℥iv.

M.

I am particular to obtain a camel's-hair brush, of good size, with long hair, so that the mixture may be well applied, being particular to introduce it far into the ear. After a few days, I usually increase the quantity of creosote as occasion may require, often using it as strong as one part of creosote to three of the oil of almonds. In pursuing this course, I have never found it produce any unpleasant symptoms, but an agreeable sensation of warmth. The duration of treatment, of course, varies according to circumstances, from three weeks to three months. While using the creosote, I syringe into the ears, every other day, the solution of soap and cologne; and, in the majority of cases use derivations behind the ears; also, occasionally, some general directions are necessary.

Looking over my note book, I find a number of interesting cases, but I will mention only two or three. About a year ago, Mrs. E., of New York, aged about 45 years, on a visit to this city, informed me that about six years previous, she found her hearing gradually failing—knew of no assignable cause. When I saw her, it was with great difficulty I could make her hear, although I spoke very loud, and she used a trumpet. Yet she told me, that when riding over the pavements in a carriage, she could hear as well as any one. This being a case of "paracusis perversæ," and of rare occurrence, I immediately became interested. I accordingly invited the lady to ride with me, and soon found that when we were travelling very quick, and the carriage making much noise, her hearing was very acute; indeed she could hear better than I could. As the lady had submitted to various modes of treatment, without any good resulting, I thought it to be a fair chance to try the creosote, and I was pleased, in a short time, to find it had given tone to the debilitated organs, and improved the hearing rapidly, so that in four months she could hear as well as before the attack.

Last April I was called to see Mrs. H. of New Hampshire, who had come to this city for medical advice. I learned from her that about ten years previous she had been attacked with a disease of the spine, and as she convalesced, her hearing gradually failed, so that at the time I saw her, she used an ear trumpet, and then could only hear when I spoke in a very loud tone. I examined the case, and found the deafness was in consequence of a want of action in the ceruminous glands. I immediately commenced the treatment before mentioned, and in about three weeks, on calling to see her one morning, she met me, "with joy sparkling in her eyes," and informed me that she had that morning heard the ticking of her watch, the first time for six years: in six weeks she attended church and could perfectly understand the clergyman, without the ear trumpet, which was to her a source of infinite satisfaction, as she was a remarkably pious lady.

A gentleman, teacher in one of the public schools, has, under my directions, been using the creosote for a few weeks, and has quite recovered his hearing: he was about relinquishing his school, when he applied to me, in consequence of his deafness, which had been getting worse for the last three or four years.

I might mention many more cases but these I think sufficient. It is of course not expected that the creosote will be of any benefit in those cases where there is mal-formation, or total obliteration of the Eustachian tube; but these cases are of rare occurrence, compared with those I have mentioned.

*Philadelphia, May* 20, 1840.

